# Medicinal Materials DNA Barcode Database (MMDBD) version 1.5—one-stop solution for storage, BLAST, alignment and primer design

**DOI:** 10.1093/database/bay112

**Published:** 2018-10-18

**Authors:** Tin-Hang Wong, Grace Wing-Chiu But, Hoi-Yan Wu, Stacey Shun-Kei Tsang, David Tai-Wai Lau, Pang-Chui Shaw

**Affiliations:** 1 Li Dak Sum Yip Yio Chin R&D Centre for Chinese Medicine, The Chinese University of Hong Kong, Hong Kong, China; 2 Institute of Chinese Medicine, The Chinese University of Hong Kong, Hong Kong, China; 3 Shiu-Ying Hu Herbarium, The Chinese University of Hong Kong, Hong Kong, China; 4 School of Life Sciences, the Chinese University of Hong Kong, Hong Kong, China

## Abstract

Authentication of medicinal materials by deoxyribonucleic acid (DNA) technology is gaining popularity. In 2010, our team has created Medicinal Materials DNA Barcode Database (MMDBD) version 1.0 to provide an interactive database for documenting DNA barcode sequences of medicinal materials. This database now contains DNA barcode sequences of medicinal materials listed in the *Chinese Pharmacopoeia*, Dietary Supplements Compendium and Herbal Medicine Compendium of the US Pharmacopoeia and selected adulterants. The data archive is regularly updated and currently it stores 62 011 DNA sequences of 2111 medicinal materials. Our team has recently completed the major improvement on the interfaces and incorporated essential bioinformatics tools to facilitate the authentication work. MMDBD version 1.5 contains detailed information of each medicinal material including their material names, medical part, pharmacopeia information, biological classification in rank of family and status on the Convention on International Trade in Endangered Species of Wild Fauna and Flora and the International Union for Conservation of Nature’s Red List of Threatened Species, if any. DNA sequences can be retrieved by search in Latin scientific name, Chinese name, family name, material name, medical part and simplified Chinese character stroke. A `BLAST’-based engine for searching DNA sequences is included in the MMDBD version 1.5. Since primer design is a key step in DNA barcoding authentication, we have integrated the `Clustal Omega alignment tool’ and `Primer3’ in the form of web interface. These new tools facilitate multiple sequence comparison and the design of primers for amplification of a target DNA barcode region, allowing DNA barcoding authentication.

## Introduction

Herbal materials have been used by many cultures for thousands of years. With the rise of the trend of natural living, the use of herbal materials as medicine and/or dietary supplements becomes more popular. The output of Chinese materia medica amounted to CNY 125.9 billion in 2013, with a 26.9% increase from the previous year (National Development and Reform Commission of China, 2014. http://www.ndrc.gov.cn/fzgggz/gyfz/gyfz/201402/t20140220_794796.html). In the USA, the total sale of herbal dietary supplements in 2016 have increased by 7.7% to USD 7.452 billion (1). The 2015 edition of *Chinese Pharmacopoeia* includes 2598 monographs about medicinal materials. Correct identification is of paramount importance for the safety and efficacy of consumption. Use of misidentified/adulterant herbs has been known to cause poisoning, including liver injury (2) and aristolochic acid nephropathy (3–4).

DNA barcoding is a popular DNA-based botanical identification method. In 2016, New York attorney general reached an agreement with several dietary supplement manufacturers to ensure that DNA barcoding and other reforms would be adopted by the manufacturers to improve quality control (New York State Office of the Attorney General, 2016. https://ag.ny.gov/press-release/ag-schneiderman-announces-major-nationwide-agreement-nbty-herbal-supplement-maker). DNA barcode is a short segment of DNA that can be used to differentiate one species from another based on sequence variation among different species. Mitochondrial cytochrome c oxidase subunit 1 (COI) is regarded as the standard barcode for animals (5–6). The Consortium for the Barcode of Life Plant Working Group has chosen the 2-locus combination of rbcL and matK as the barcode for land plants (7) after comparing seven candidates for their universality (amplification success and sequencing success), sequence quality and power of species discrimination. Nevertheless, these two regions do not have enough discriminatory power for closely related plant groups (8). Chen and colleagues proposed the second internal transcribed spacer (ITS2) between ribosomal RNA genes as another DNA barcode for medicinal plants, as ITS2 sequence could successfully discriminate 92.7% of the tested samples at species level (9).

At present, the Barcode of Life Data System (http://v3.boldsystems.org/) is a workbench for accessing and use of DNA barcoding data for animals (COI), plant (matK+rbcL) and fungi (ITS) (10). Chen and colleagues constructed an online DNA barcoding database for traditional medicines (http://www.tcmbarcode.cn/china) that selected ITS2 and psbA–trnH for medicinal plants and COI for medicinal animals (11). Both databases allow online identification by similarity search. In 2010, our group launched Medicinal Materials DNA Barcode Database (MMDBD version 1.0), the first of its kind, that collected rbcL, matK, nuclear ribosomal ITS, ribosome DNA and other plastid NDA regions (12). Now, MMDBD version 1.0 have been significantly enhanced into MMDBD version 1.5, a web-based, one-stop platform that allows sequence access, sequence similarity search, multiple sequence alignment and primer design. Our objectives are (i) to expand the existing database to cover the sequences of more medicinal materials and adulterants; (ii) to enhance the efficiency on sequence access, sequence similarity search, multiple sequence alignment and primer design and to work out these procedures in one platform; and (iii) to increase the accuracy for sequence alignment and primer design by reducing the copying times when transferring the sequence between different platforms. Total number of species included in MMDBD version 1.5 has increased from 1259 to 2111, with the number of sequences increased from 18 436 to 62 011. With these sequences archived, the database can be used as the first-step tool for identifying a barcode sequence from an unknown medicinal material, especially in screening suspected medicinal materials.

## Materials and methods

### Data collection

Medicinal species listed in the *Pharmacopoeia of the People’s Republic of China* (2015 edition) (13), American Herbal Pharmacopoeia (http://www.herbal-ahp.org), Dietary Supplements Compendium and Herbal Medicines Compendium of the United States Pharmacopeia (14) as well as prescriptions of folk medicine and selected adulterants and closely related species were included in the database. DNA regions included for plants are chloroplast matK and rbcL, nuclear ITS, nuclear ribosomal RNA and other chloroplast DNA regions. For animals, COI and other mitochondrial DNA regions were included. For fungi, nuclear ITS sequences were collected. Sequences were retrieved from INSD-Seq eXtensible Markup Language (XML) files from GenBank. Data were extracted from the XML files by scripts and irrelevant sequences, such as shotgun sequences, microsatellites and Inter-Simple Sequence Repeat (ISSR) markers, were filtered.

### Implementation

In MMDBD version 1.5, an open source database system MariaDB (version 10.1.18) was used as the related database. NCBI BLAST (v 2.2.26) was used as sequence similarity search engine. With Perl DBI (Database independent interface) module connecting the SQL-based MariaDB, BLAST results are obtained through picking up stored query tasks by background job assigned through Perl interface. BLAST query was through PHP Hypertext Preprocessor, a general-purpose server side scripting language for displaying dynamic web pages, post request and getting the corresponding response XML file that was generated by PHP executing NCBI BLAST engine. After the conversion of the responded XML into JSON (JavaScript Object Notation) format, results are displayed as hit chart by JavaScript XML library.

Clustal Omega version 1.020 and Primer3 version 4.0 were implemented with the same machine of the BLAST engine for direct application of the results from BLAST. The queries of the Clustal Omega and Primer3 were submitted through HTML post to CGI execution. Direct responses were in the format of HTML and displayed within the framework of the MMDBD.

The previous version 1.0 database used NCBI BLAST version 2.2.24 with a dual core 1.6 GHz CPU, Central Processing Unit (main processor) of the server. Based on a 500-character BLAST search, it required >150 s to give out the results on average. In the current version, NCBI BLAST version 2.2.26 with a 6-core 1.6 GHz CPU and 8 GB RAM (Random Access Memory) for up to six threads was introduced. For the same 500-character BLAST search, it only required 7 s to give the results on average. The usability has been greatly advanced.

## Results and discussion

### Data access

The sequences can be accessed by clicking a species on the full species list of the home page, or by searching the species’ Latin name, material name, simplified Chinese name or the number of strokes of the first Chinese word of the simplified Chinese name. Detailed information of the medicinal materials will be displayed by clicking on the sequence line. The information includes the material name, medical part, pharmacopeia information, family (taxonomic rank), adulterants, photos and status on the Convention on International Trade in Endangered Species of Wild Fauna and Flora and the International Union for Conservation of Nature’s Red List of Threatened Species.

### Workflow for the integrated DNA analysis functions

**Figure 1:**
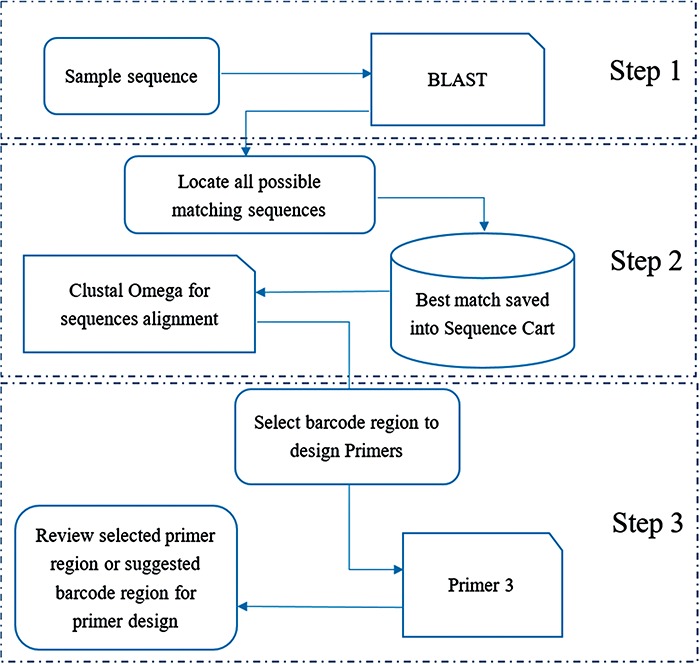
General workflow of `one-stop, three steps approach’ for designing primer for barcoding.

A `one stop, three steps approach’ was developed in the MMDBD version 1.5 to facilitate the transaction of sequences among three major tools, `BLAST’, `Clustal Omega’ and `Primer3’ for designing polymerase chain reaction primer (Figure 1). For Step 1, a sample sequence is searched in the built-in BLAST system against all sequences in the MMDBD. In Step 2, the best match sequence can be saved into an intermediate sequence storage named `Sequence Cart’, which is generated by the web server automatically for each visitor. In the Alignment & Primer Design page, users can then import the selected sequences and process an alignment with `Clustal Omega’. Finally, in Step 3, users can pick their own primer for amplification based on the alignment results with the built-in `Primer3’ or receive suggested primers from that.

The integrated platform allows users to get the three major primer-designing processes done in one web site, avoiding the possible errors from repeatedly copying and pasting the sequence in various open-source engines.

### Operating procedures

**Figure 2:**
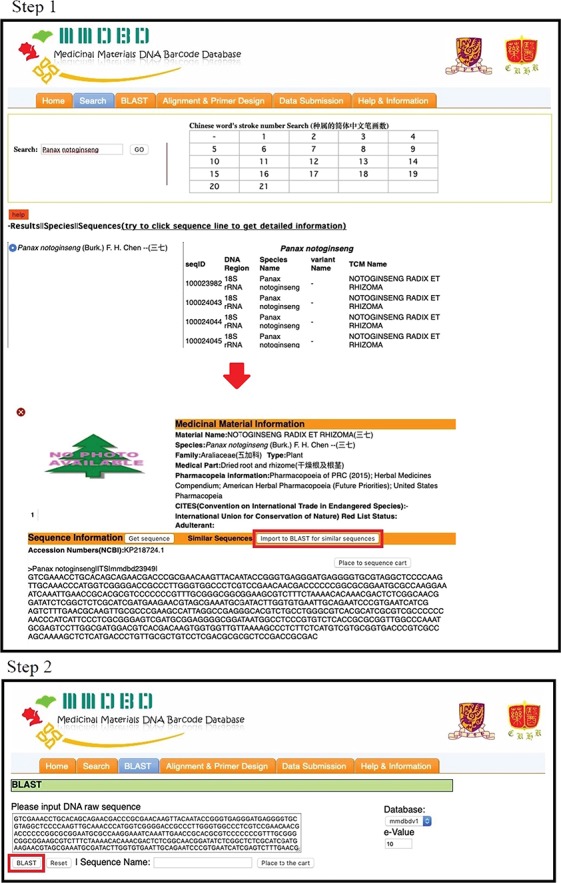
The operating procedures of primer design on the one-stop platform—sequence search and BLAST.

**Figure 3:**
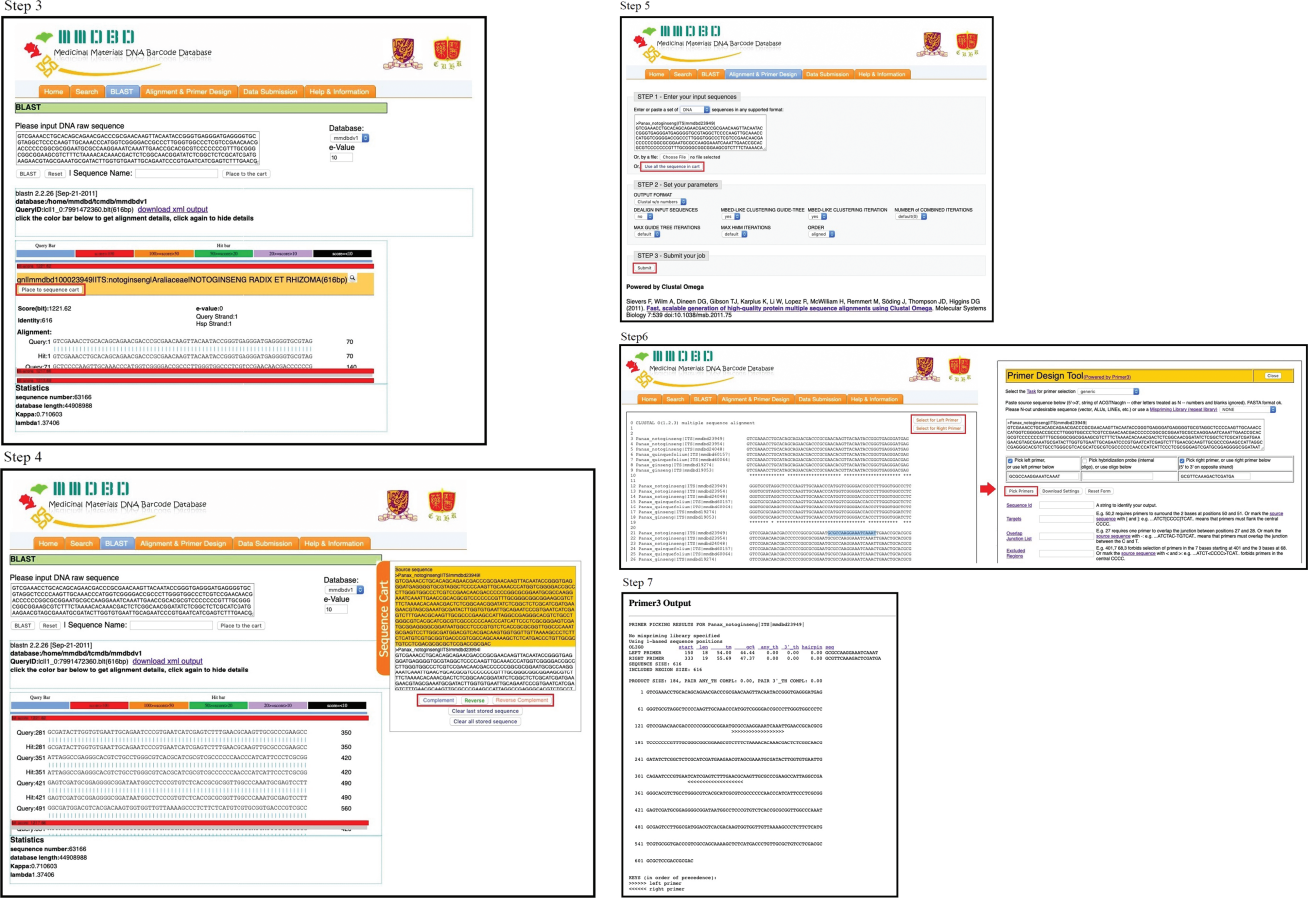
**(1)** The operating procedures of primer design on the one-stop platform—selection of blasted sequences into sequence cart. **(2)** The operating procedures of primer design on the one-stop platform—multiple sequence alignment and primer design.

In order to perform molecular authentication, it is important to check for the presence of forensically informative nucleotides (FINs). FINs are species-specific nucleotides in a certain DNA region and can be used to discriminate the species from its common adulterants or closely related species. For some medicinal materials with high economic value or that are commonly adulterated, their FINs, also called nucleotide signatures, have already been published by different groups of scientists (15–17). For most species, it is still necessary to identify the DNA region(s) with FINs by aligning different DNA regions of the target species and the adulterants/closely related species before carrying out any `wet lab’ experiment. MMDBD version 1.5 allows users to find similar sequences of closely related species by in-house BLAST and temporarily save selected sequences in a sequence cart for alignment. The operating procedures are illustrated in Figure 2 (Steps 1 and 2), Figure 3(1) (Steps 3 and 4) and Figure 3(2) (Steps 5 and 7) with *Panax notoginseng* as an example.

***Steps 1 and 2.***Under the Search page, user can find the sequences of the species of interest. By clicking on the sequence of a certain DNA region, ITS in this example detailed information of the medicinal material as well as the sequence is shown. User can submit the sequence to BLAST search by clicking on the `Import to BLAST for similar sequences’ button.

***Steps 3 and 4***. BLAST search result entries are displayed below the query box. User can click on each result entry to view the sequence information and add the sequences into the `Sequence Cart’. Here, three sequences from *P*. *notoginseng*, *P*. *quinquefolium*, *P*. *ginseng* and *P*. *japonicus* were added to Sequence Cart. User may add more sequences from suspected adulterants that are not closely related to the target species, and therefore are not likely to be matched by BLAST, into the Sequence Cart for alignment. The Sequence Cart can be viewed by clicking the orange `Sequence Cart’ on the right. Sequences added to the Sequence Cart can be individually converted to complement, reverse or reverse complement sequences.

***Step 5.*** Under the Alignment & Primer Design page, all sequences in cart are submitted for alignment with Clustal Omega by clicking `use all the sequence in cart’ button and the `Submit’ button.

***Step 6.*** From the alignment output by Clustal Omega, user can look for the FINs of *P*. *notoginseng*. Sometimes, a few sequences may be stored as the opposite strand, i.e. in reverse complement, and cannot be aligned. If this happens, just go back to the Sequence Cart and carry out reverse complement manipulation and align them again. If FINs of the target species can be found, user may try to sequence that DNA region with universal primers, if available. Or they can design species-specific primers with the FINs on the 3′′ end of primers or sequencing primers that flank the FINs to allow identification by sequencing (18). This can be easily done by highlighting the potential primer sequence and clicking on the button `Select for Left Primer’ or `Select for Right Primer’ to transfer the highlighted DNA sequence to the interface of the Primer3. The first DNA entry in the alignment will also be automatically loaded to Primer3 as DNA template. To select the sequence for the opposite primer, close the Primer3 pop-up and highlight the chosen sequence and click the `Select for Left (or Right) Primer’ button. Then both primer sequences will be entered to Primer3.

***Step 7.*** Primer3 output can be reviewed.

## Concluding remarks

MMDBD version 1.5 has a significant increase in the number of medicinal materials and their DNA sequences listed in the *Chinese Pharmacopoeia*, American Herbal Pharmacopoeia and Dietary Supplement Compendium and Herbal Medicine Compendium of the United States Pharmacopeia. With the enhancement of the CPU power and System software integration, MMDBD version 1.5 provides a quick solution for medicinal material identification. Speedy BLAST search results and window switching-free interfaces would avoid the errors from the data transaction manually among different data processing engines. This implies reducing errors and working time.

## Availability and requirements

The MMDBD version 1.5 is publicly available and can be accessed through http://rdccm.cuhk.edu.hk/mherbsdb/.

The database may be browsed with Internet Explorer version 11, Chrome version 48 and Firefox version 48 or above.

## Notes

**Database URL**: http://rdccm.cuhk.edu.hk/mherbsdb/

## References

[1] Smith T., Kawa K., Eckl V. et al. (2017) Herbal supplement sales in US increase 7.7% in 2016. *Herbalgram*, 115, 56–65.

[2] Teschke R., Zhang L., Long H. et al. (2015) Traditional Chinese medicine and herbal hepatotoxicity: a tabular compilation of reported cases. *Ann. Hepatol.*, 14, 7–19. 25536637

[3] Debelle F.D., Vanherweghem J.L. and Nortier J.L. (2008) Aristolochic acid nephropathy: a worldwide problem. *Kidney Int.*, 74, 158–169. 18418355 10.1038/ki.2008.129

[4] Lo S.H., Mo K.L., Wong K.S. et al. (2004) Aristolochic acid nephropathy complicating a patient with focal segmental glomerulosclerosis. *Nephrol. Dial. Transplant.*, 19, 1913–1915. 15199198 10.1093/ndt/gfh159

[5] Hebert P.D., Ratnasingham S. and deWaard J.R. (2003) Barcoding animal life: cytochrome c oxidase subunit 1 divergences among closely related species. *Proc. Biol. Sci.*, 270, S96–S99. 12952648 10.1098/rsbl.2003.0025 PMC1698023

[6] Dawnay N., Ogden R., McEwing R. et al. (2007) Validation of the barcoding gene COI for use in forensic genetic species identification. *Forensic Sci. Int.*, 173, 1–6. 17300895 10.1016/j.forsciint.2006.09.013

[7] CBOL Plant Working Group (2009) A DNA barcode for land plants. *Proc. Natl. Acad. Sci. USA*, 106, 12794–12797. 19666622 10.1073/pnas.0905845106 PMC2722355

[8] Zhang C.Y., Wang F.Y., Yan H.F. et al. (2012) Testing DNA barcoding in closely related groups of Lysimachia L. (Myrsinaceae). *Mol. Ecol. Resour.*, 12, 98–108. 21967641 10.1111/j.1755-0998.2011.03076.x

[9] Chen S., Yao H., Han J. et al. (2010) Validation of the ITS2 region as a novel DNA barcode for identifying medicinal plant species. *PLoS One*, 5, e8613. 20062805 10.1371/journal.pone.0008613 PMC2799520

[10] Ratnasingham S. and Hebert P.D. (2007) bold: the Barcode of Life Data System (http://www.barcodinglife.org). *Mol. Ecol. Notes*, 7, 355–364. 18784790 10.1111/j.1471-8286.2007.01678.x PMC1890991

[11] Chen S., Pang X., Song J. et al. (2014) A renaissance in herbal medicine identification: from morphology to DNA. *Biotechnol. Adv.*, 32, 1237–1244. 25087935 10.1016/j.biotechadv.2014.07.004

[12] Lou S.K., Wong K.L., Li M. et al. (2010) An integrated web medicinal materials DNA database: MMDBD (Medicinal Materials DNA Barcode Database). *BMC Genomics*, 11, 402. 20576098 10.1186/1471-2164-11-402 PMC2996930

[13] The Pharmacopoeia Editorial Committee (2015) *Pharmacopoeia of the People’s Republic of China*. Chemical Technology Press, Shanghai, China.

[14] United States Pharmacopeial Convention (2016) *United States Pharmacopeia*. United States Pharmacopeial Convention, Rockville, MD.

[15] Liu Y., Wang X., Wang L. et al. (2016) A nucleotide signature for the identification of American ginseng and its products. *Front. Plant Sci.*, 7, 319. 27047504 10.3389/fpls.2016.00319 PMC4796032

[16] Wang X., Liu Y., Wang L. et al. (2016) A nucleotide signature for the identification of Angelicae Sinensis Radix (Danggui) and its products. *Sci. Rep.*, 6, 34940. 27713564 10.1038/srep34940 PMC5054691

[17] Gao Z., Liu Y., Wang X. et al. (2017) Derivative technology of DNA barcoding (nucleotide signature and SNP double peak methods) detects adulterants and substitution in Chinese patent medicines. *Sci. Rep.*, 7, 5858. 28724933 10.1038/s41598-017-05892-y PMC5517575

[18] Li M., Zhang K.Y.B., But P.P.H. et al. (2011) Forensically informative nucleotide sequencing (FINS) for the authentication of Chinese medicinal materials. *Chin. Med.*, 6, 42. 22153058 10.1186/1749-8546-6-42 PMC3253680

